# Renal Pathologic Findings in TAFRO Syndrome: Is There a Continuum Between Thrombotic Microangiopathy and Membranoproliferative Glomerulonephritis? A Case Report and Literature Review

**DOI:** 10.3389/fimmu.2019.01489

**Published:** 2019-06-28

**Authors:** Amélie Leurs, Viviane Gnemmi, Arnaud Lionet, Loïc Renaud, Jean-Baptiste Gibier, Marie-Christine Copin, Eric Hachulla, Pierre-Yves Hatron, David Launay, David Fajgenbaum, Louis Terriou

**Affiliations:** ^1^Département de Médecine Interne et Immunologie Clinique, CHU Lille, Centre de Référence des Maladies Auto-Immunes Systémiques Rares du Nord et Nord-Ouest de France (CeRAINO), LIRIC INSERM U995, Université de Lille, Lille, France; ^2^CHU Lille, Institut d'Immunologie, Université de Lille, Lille, France; ^3^Département d'Anatomo-Cyto-Pathologie, CHU Lille, Centre de Biologie Pathologie, Université de Lille, Lille, France; ^4^Département de Néphrologie, CHU Lille, Université de Lille, Lille, France; ^5^Département des Maladies du Sang, CHU Lille, Université de Lille, Lille, France; ^6^Department of Translational Medicine and Human Genetics, University of Pennsylvania, Philadelphia, PA, United States

**Keywords:** TAFRO syndrome, idiopathic multicentric Castleman disease, membranoproliferative glomerulonephritis, thrombotic microangiopathy glomerulopathy, VEGF, interleukin-6

## Abstract

**Background:** TAFRO syndrome is a clinical subtype of idiopathic multicentric Castleman disease (iMCD) that is characterized by thrombocytopenia, anasarca, fever and/or elevated serum C-reactive protein, renal dysfunction, and organomegaly.

**Case Presentation:** A 28-year-old woman with fever, weight gain of 13 kgs, lower extremity edema, hepatosplenomegaly, and multicentric peripheral lymphadenopathy was referred to our center. Laboratory investigations revealed anemia, thrombocytopenia, creatinine at 1.19 mg/dL and hypoalbuminemia at 33 g/L. Proteinuria was measured at 2 g/day including albuminuria at 1.5 g/day. Urinary sediment examination found leukocyturia at 44,000/mL and hematuria at 645,000/mL. Vascular endothelial growth factor (VEGF) level was elevated. A cervical lymph node biopsy found features consistent with the mixed histopathological subtype of iMCD. A renal biopsy revealed a membranoproliferative glomerulonephritis (MPGN) pattern. We initiated 3 days of methylprednisolone pulse-therapy at 1,000 mg per day, followed by prednisone 1 mg/kg/day and evolution was favorable.

**Review of Literature:** 19 iMCD patients with TAFRO syndrome had undergone a renal biopsy: 8 cases with author's diagnosis consistent with MPGN-like and 11 cases of thrombotic microangiopathy (TMA)-like glomerulopathy without fibrin thrombi in glomerular capillaries or arterioles and without typical biological signs. Clinical, biological, and outcome characteristics were similar between the cases described as having MPGN and TMA-like presentation. After a thorough review of histopathological descriptions for each case, MPGN lesions seems to be the consequences of chronic glomerular endothelial injury in persistent TMA. We suspect that VEGF and IL-6 play a key role in the physiopathology of the spectrum of renal involvement from TMA-like to MPGN observed in TAFRO syndrome.

**Conclusion:** We present a Caucasian iMCD patient with TAFRO syndrome with renal insufficiency secondary to MPGN, which might be secondary to a chronic TMA-like disease. We suspect that there is a continuum between TMA and MPGN lesions in TAFRO syndrome favored by VEGF and IL-6.

## Background

TAFRO syndrome describes a clinical subtype of idiopathic multicentric Castleman disease (iMCD) that is characterized by thrombocytopenia, anasarca, fever and/or elevated serum C-reactive protein, renal dysfunction or reticulin myelofibrosis, and organomegaly (hepatomegaly, splenomegaly, lymphadenopathy) (1, 2). Patients often also demonstrate characteristic Castleman disease histopathology in multiple regions of enlarged lymph nodes, elevated alkaline phosphatase, reticulin myelofibrosis, and an elevated number of megakaryocytes in bone marrow (BM) (2). Kidney dysfunction is frequently observed in patients with TAFRO syndrome but the underlying mechanisms are poorly understood (3) as renal histopathology has been rarely described. Here, we report on a European female with the TAFRO syndrome subtype of iMCD and renal histology consistent with membranoproliferative glomerulonephritis (MPGN); we review the literature of all renal biopsy cases described to our knowledge.

## Case Presentation

A 28-year-old woman without significant past medical history was referred to our institution for menorrhagia and 1 month history of progressive malaise (ECOG performance status: 1). She was treated with oral estro-progestative contraception at the time. Physical examination found fever, hypertension, asthenia, anorexia, dyspnea, abdominal discomfort, weight gain of 13 kgs, lower extremity edema, hepatosplenomegaly, and multicentric peripheral lymphadenopathy.

Laboratory investigations revealed normocytic, non-regenerative anemia (hemoglobin: 6.1 g/dL), thrombocytopenia at 24 × 10^9^/L, leukocytosis at 14.3 × 10^9^/L, with myelocytosis at 3% and dacryocytes on blood smear, blood urea nitrogen at 15.4 mg/dL, creatinine at 1.19 mg/dL (estimated glomerular filtration rate at 62 ml/min/1.73 m^2^ according to CKD-EPI formula), hypoalbuminemia at 33 g/L, cholestasis (alkaline phosphatase at 314 U/L, gamma glutamyltransferase at 369 U/L) without cytolysis, elevated fibrinogen at 7.8 g/L, elevated C-reactive protein level at 150 mg/L and, elevated β2 microglobulin level at 3.94 mg/L. Haptoglobin test and bilirubin levels were normal. Proteinuria was measured at 2 g/day including albuminuria at 1.5 g/day. Renal echography was normal. Urinary sediment examination found leukocyturia at 44,000/mL and hematuria at 645,000/mL but no bence-jones proteinuria. The blood protein electrophoresis showed an inflammatory profile without monoclonal bands on immunofixation test (total gamma globulins 13 g/L). There was no immunophenotypic aberrancy of lymphocytes by flow cytometry analysis (normal kappa/lambda ratio). Molecular studies were negative for breakpoint cluster region-abelson-1 fusion as well as, janus kinase 2 (JAK2), thrombopoietin receptor (MPL), and calreticulin (CALR) mutations. Auto-immune (complement, cryoglobulinemia, rheumatoid factor, anti-glomerular basement membrane, anti-neutrophil cytoplasmic, antinuclear antibodies) and viral [hepatitis C and B, Epstein-Barr virus (EBV), cytomegalovirus, parvovirus B19, Human Herpesvirus (HHV)-6 and -8, human immunodeficiency virus and syphilis] tests were negative. Vascular endothelial growth factor (VEGF) level was elevated at 640 pg/mL (normal range: < 500 pg/mL).

Computerized Tomography-scan showed diffuse lymphadenopathy, pleural and peritoneal effusion, and hepatosplenomegaly. Positron emission tomography found hypermetabolic lymph nodes, spleen and BM. The BM trephine biopsy evaluation revealed hyperplasia of the myeloid, erythroid, and megakaryocytic series without maturation anomalies, an overall myelofibrosis grade 2 using European consensus on bone marrow fibrosis grading, and polytypic plasmacytosis at 10%, without blast cells. Cytogenetic analysis of the BM aspirate demonstrated a diploid female karyotype.

**Figure 1:**
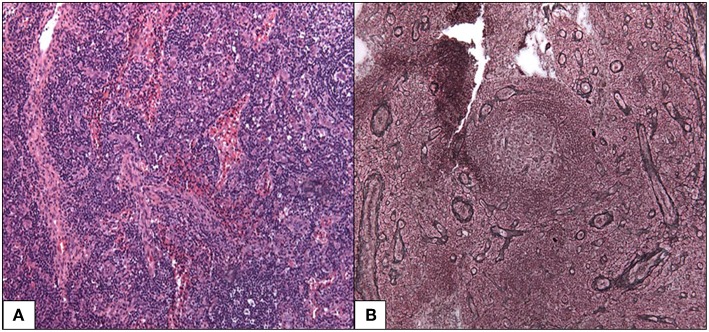
Mixed histopathological subtype of Castleman disease. **(A)** Follicles are characterized by an atrophic germinal center with few lymphocytes, radially penetrated by blood vessels (lollipop follicle). HES 100x **(B)** the small lymphocytes of the mantle zones are arranged in concentric rings around the germinal center. Silver impregnation after Gordon-Sweet 100x.

A cervical lymph node biopsy found features consistent with the mixed histopathological subtype of iMCD (Figure 1). Follicles were characterized by atrophic germinal centers with few lymphocytes and radially penetrating blood vessels (lollipop finding). The small lymphocytes of the mantle zones were arranged in concentric rings around the germinal center (onion skinning). There were also increased vessels and abundant plasma cells in the interfollicular region. We noted absence of Kappa or Lambda monotypic immunoglobulin and negativity for HHV-8 and EBV encoded RNA probe.

**Figure 2:**
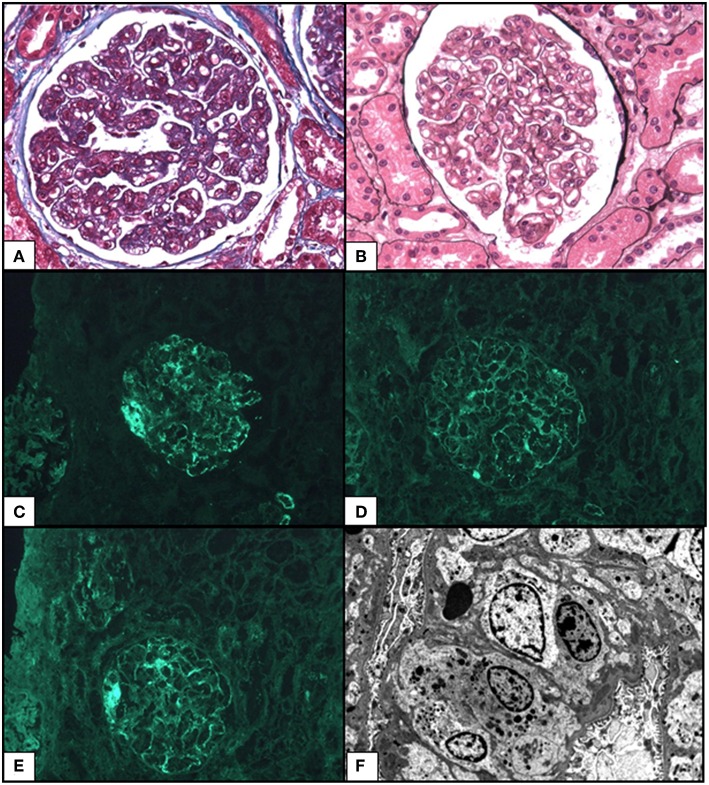
Representative photomicrographs from kidney biopsy of the case: membranoproliferative glomerulonephritis pattern. **(A)** Increased lobulation with intracapillary hypercellularity by macrophages and neutrophils. Masson's Trichrome 400x. **(B)** Thickening of capillary due to multiple double contours. Mild mesangial proliferation. Silver impregnation after Jones 400x. Mild. **(C)** Segmentary granular capillary walls deposits for IgM. Immunofluorescence (IF) 400x. **(D)** Mild endomembranous deposits for Lambda light chains. IF 400x. **(E)** Mild endomembranous deposits for Kappa light chains. IF 400x. **(F)** Ultrastructural study showed moderate subendothelial dense deposits without subepithelial neither intra-membranous deposits x1293.

A renal biopsy revealed 32 glomeruli a MPGN pattern was observed with endocapillary proliferation (macrophages and neutrophils), mild mesangial proliferation, mesangiolysis, endothelial cell swelling and thickening of capillaries due to multiple double contours (light microscopy) (Figure 2). There is also one focal segmental glomerulosclerosis lesion (tip variant). No thrombosis, no interstitial and tubular involvement, and no evidence of vasculitis were observed, but arteriosclerosis was present. Immunofluorescence found bulky endomembranous segmentary deposits for IgM, mild deposits for C1q, kappa and lambda, and no deposits for IgG, IgA and C3; this pattern was consistent with polytypic MGPN. An ultrastructural study revealed discrete electron-dense deposits without organization in the subendothelial space, partial podocyte foot process effacement, and absence of sub-epithelial or intra-membranous deposits. Considering (i) negative cryoglobulinemia, negative antinuclear antibodies, normal complement, (ii) mesangiolysis, endothelial cell swelling, mild mesangial proliferation, and multiple double contours, (iii) endomembranous focal deposits, the MPGN-like seems to occur in the settings of a chronic TMA.

Considering the clinical, laboratory, and histopathological findings, the diagnosis of the TAFRO syndrome [grade 3 severity, (2)] subtype of iMCD (4) was made, and 1 mg/kg of prednisone was initiated. As we did not observe any laboratory improvement, we initiated 3 days of methylprednisolone pulse-therapy at 1,000 mg per day. We then observed a normalization of platelets, CRP, creatinine serum level (0.84 mg/dL), and proteinuria. Nine months later, the patient was in a complete remission and we progressively tapered the corticosteroid treatment. The patient remains in complete remission off of all therapy for 2 years, and she was able to give birth to a healthy child without recurrence of the disease.

## Review of Literature

The renal biopsy findings of polytypic MGPN on TMA in this patient led us to search the literature for other patients with TAFRO syndrome that had undergone a renal biopsy. A comprehensive literature review of all published cases of TAFRO syndrome, found 19 patients who had undergone a renal biopsy (Table 1) to the best of our knowledge. There were 8 males and 17 Asians (2 Caucasian); the median age at diagnosis of TAFRO was 69 years [53; 76]. Serum interleukin-6 (IL-6) and VEGF level, when available, were elevated in all but one case (13). The median creatinine level at the time of renal biopsy was 2.13 mg/dL [1.25; 2.52], and urinary sediment was present in most cases (glomerular hematuria in 13 cases and proteinuria over 1 g per gram of creatinine or per day in 7 cases). Two cases were described as having nephrotic syndrome (5, 14). Nine cases (47%) presented with severe renal insufficiency requiring hemodialysis and 11 cases (58%) needed treatment beyond corticosteroid monotherapy to induce disease response (e.g., plasma exchange, tocilizumab, intravenous immunoglobulin, and rituximab). Hemodialysis could be discontinued in 7 out of 9 cases following appropriate therapy, one patient died and only one patient needed long-term hemodialysis for an end-stage renal disease (3). In total, five patients (26%) died, including 4 patients who had benefited from hemodialysis. The etiology of the death was unknown for 2 cases, a cerebral hemorrhage was responsible for 1 case, gastrointestinal bleeding for 1 case, and sepsis for the last one.

**Table 1:** All reviewed cases of TAFRO syndrome with renal histology.

**References**	**Sex**	**Age**	**Ethnicity**	**HHV8/HIV**	**IL-6 (pg/mL)**	**VEGF (pg/mL)**	**Serum creatinine (mg/dL)**	**GH**	***P***	**Outcome**			**Renal histopathology described by authors**													**Underlying etiology established after histopathological review**
										**HD**	**Death**	**TTT**	**Glomeruli number**	**EnP**	**Endothelial cell swelling**	**ExP**	**Glomerulo sclerosis**	**Thickening of basement membrane**	**MP**	**Double contours of GBM**	**Mesangiolysis**	**IF (glomerulocapillary) deposits**	**Electron dense deposits**	**Interstitial fibrosis and tubular atrophy**	**Conclusion by authors**	
Our case	F	28	Caucasian	N	na	640	1.19	Y	1.58 g/d	N	N	CS	32	Y	Y	N	Y	Y	Y	Y	Y	1 +	Y	N	MPGN-like	TMA compatible
Ito et al. (5)	F	76	Asian	N	49.2	1,350	3.02	Y	0.30 g/d	Y	N	CS	29	Y	N	N	Y	Y	Y	Y	N	0 +	N	Y	MPGN-like	TMA compatible
Hashimoto et al. (6)	M	69	Asian	na	26.6	183	2.22	Y	0.25 g/d	Y	Y	CS, ciclosporin	na	Y	N	N	N	Y	Y	Y	N	3 +	Y	N	MPGN-like	MPGN with deposits
Furuto et al. (7)	F	55	Asian	N	11.7	464	0.89	Y	2.3 g/g	N	N	CS	11	N	N	N	N	Y	Y	Y	N	0 +	N	Y	MPGN-like	TMA compatible
Tanaka et al. (8)	M	70	Asian	N	33	126	5.48	N	0.33 g/d	Y	N	CS, eltrombopag	55	N	Y	N	Y	Y	N	Y	Y	0 +	N	N	MPGN-like	TMA compatible
Kawashima et al. (9)	M	38	Asian	N	na	4,420	2.58	Y	Y	N	N	CS	na	N	N	N	N	Y	Y	Y	N	2 +	na	N	MPGN-like	MPGN with deposits
Hamada et al. (10)	F	77	Asian	na	na	na	0.83	Y	Y	N	N	CS	na	na	na	na	Na	na	na	na	na	na	na	na	MPGN-like	na
Tsukada et al. (11)	M	69	Asian	na	na	na	2.81	na	1.7 g/d	Y	Y	CS, PE	na	na	na	na	Na	na	na	na	na	na	na	na	MPGN-like	na
Kakeshita et al. (12)	F	69	Asian	na	na	na	1.20	na	4 g/g	N	Y	CS, ciclosporin	na	na	na	na	Na	na	na	na	na	na	na	na	MPGN-like	na
Louis et al. (3)	M	67	Caucasian	N	22	Elevated	5.24	Y	5.29 g/L	Y	N	CS, Toci, RTX	na	N	N	N	N	Y	Y	N	N	0 +	na	Y	TMA-like GM	TMA compatible
Noda et al. (13)	F	79	Asian	N	3.76	15.6	1.86	Y	2.65 g/g	Y	N	CS, PE, RTX	5	N	N	N	N	Y	N	Y	Y	0 +	N	N	TMA-like GM	TMA compatible
Nakamori et al. (14)	F	54	Asian	N	8,2	na	1.66	Y	4.2 g/d	N	N	CS	23	Y	Y	N	Y	N	N	N	N	0 +	N	Y	TMA-like GM	TMA compatible
Noda-Narita et al. (15)	F	80	Asian	N	21.3	454	1.17	Y	0.41g/d	N	N	Melphalan, IVIg, CS, Toci, romiplostim	8	Y	Y	N	Y	Y	N	Y	N	1 +	N	Y (20%)	TMA-like GM	TMA compatible
Ozeki et al. (16)	F	51	Asian	na	21.2	198	1.03	N	0.52 g/g	N	N	CS	16	N	Y	Y	Y	Y	N	Y	Y	0 +	N	N	TMA-like GM	TMA compatible
Mizuno et al. (17)	M	84	Asian	na	12.3	177	2.30	Y	0.30 g/d	Y	Y	CS, PE, Toci	29	Y	Y	N	Y	N	N	N	Y	0 +	N	N	TMA-like GM	TMA compatible
Pais et al. (18)	F	31	Asian	N	na	6,680	2.36	N	0.23 g/g	N	N	CS, Toci	na	N	N	N	N	N	N	Y	Y	0 +	N	N	TMA-like GM	TMA compatible
Nakamura et al. (19)	M	76	Asian	na/N	14,1	na	1.30	na	Y	Y	Y	CS	na	N	Y	N	N	Y	Y	Y	N	0 +	N	N	TMA-like GM	TMA compatible
José et al. (20)	F	61	Caucasian	N	722.6	na	2.13	na	Na	Y	N	CS, Toci, RTX	na	N	Y	N	N	Y	Y	Y	Y	0 +	na	N	TMA-like GM	TMA compatible
Sasaki et al. (21)	F	49	Asian	na	na	na	2.45	Y	Y	N	N	CS, Toci	na	na	na	na	Na	na	na	na	na	na	na	na	TMA-like GM	na
Takahasi et al. (22)	M	34	Asian	na	na	na	1.76	Y	1.65 g/g	N	N	CS	na	na	na	na	Na	na	na	na	na	na	na	na	TMA-like GM	na

*IL, interleukin; VEGF, vascular endothelial growth factor; GH, glomerular haematuria; P, proteinuria; HD, haemodialysis; TTT, treatments; MPGN, membranoproliferative glomerulonephritis; TMA-like GM, thrombotic microangiopathy-like glomerulopathy; EnP, endocapillary proliferation; ExP, extracapillary proliferation; MP, mesangial proliferation; GBM, glomerular basement membrane; IF, immunofluorescence; F, female; M, Male; Y, yes; N, no; CS, corticosteroids; Toci, tocilizumab; RTX, rituximab; IVIg, intravenous immunoglobulin; PE, plasma exchange; HHV8, Human Herpesvirus-8; HIV, Human Immunodeficiency Virus*.

Concerning renal histology, we found 8 cases with author diagnosis consistent with MPGN and 11 cases of thrombotic microangiopathy (TMA)-like glomerulopathy without fibrin thrombi in glomerular capillaries or arterioles and without typical biological signs. We found two additional cases that described renal histological evaluation at autopsy and two others cases of MPGN but details were not available, which were not included in our description (23–25).

Clinical, biological (including renal function and urinary sediment as well as IL6 and VEGF levels), and outcome characteristics were similar between patients with MPGN and TMA-like presentation.

## Discussion

We present a typical case of the TAFRO syndrome subtype of iMCD, who met diagnostic criteria for iMCD and demonstrated all 3 major criteria and 4 minor criteria for TAFRO syndrome (2, 4). Interestingly, this patient responded well to corticosteroid monotherapy, and a renal biopsy revealed the progressive renal insufficiency was due to MPGN.

In CD, renal complications seems to be uncommon and in historical case series with renal involvement, there have been various histological diagnoses, such as secondary amyloidosis, MPGN, membranous glomerulonephritis, TMA-like lesions, mesangial proliferative glomerulonephritis, crescentic glomerulonephritis, minimal change disease, and tubulointerstitial nephritis (26, 27). TMA-like lesions have been the most commonly reported feature (55%), followed by AA amyloidosis (15%) (27). MPGN or MPGN-like were described in 11% of case (26).

However, none of these historical case series have specifically described renal histopathology in the newly reported TAFRO subtype of iMCD, and renal biopsies have been rarely reported in iMCD case reports, possibly due to thrombocytopenia making renal biopsies difficult.

As renal dysfunction is reported in more than one-half of TAFRO cases (2) and the mechanism remains unclear, we performed a literature review to characterize renal histopathology. The two patterns described in the recent literature are most consistent with MPGN (42%) and a TMA-like histology (58%) but without fibrin thrombi in glomerular capillaries or arterioles and without typical biological signs (Table 1). On light microscopy, MPGN and chronic TMA shared similar histological pattern: mesangial proliferation, double-contours and endocapillary proliferation (related to immune cells hypercellularity for MPGN and endothelial swelling for TMA), making their diagnostic complex (28, 29). This difficulty is generally overcome by the detection of immune deposits on immunofluorescence study that favor for MPGN diagnosis. However, as weak to moderate immune deposits can be accepted in TMA (30, 31), MPGN diagnosis is warranted in the settings of intense immune deposits on immunofluorescence and/or electron-dense deposits (29, 30). After a thorough review of histopathological descriptions for the 20 cases: 4 MPGN-like patients seems to be a great deal of overlap between TMA (absence of immune complex deposits), 2 patients presented MPGN with straight deposits for which it is difficult to conclude in favor of a TMA (but without it being incompatible), 9 patients presented more argument in favor of TMA and 5 patients for which the details of the renal biopsy is not available (Table 1). One previous report speculates that renal histology in the early stages of TAFRO syndrome may begin with endotheliosis and glomerular double-contours, which are consistent with histopathology of TMA (19). Since we didn't find any etiology to explain MPGN, we suspect that there is a histopathological and probably physiopathology continuum between TMA and MPGN. TMA seems to appear in first and if it becomes chronic the histopathology pattern becomes more in favor of MPGN. In our review, the descriptions of TAFRO syndrome cases having TMA-like histological examination on renal biopsies were similar to what was reported previously in iMCD (endothelial swelling, glomerular capillary-loop double contours and mesangiolysis) with predominantly glomerular involvement (27).

Among the 10 cases in the literature of TAFRO syndrome requiring hemodialysis identified by Tanaka et al., the proteinuria and hematuria both tended to be mild; nephrotic-range proteinuria was only reported in 1 case. Only 1 out of the 10 cases requiring hemodialysis died by the time of follow up (8). Yuan et al. described that when patients present with renal complications, the evolution showed an aggressive and fatal course with observed death in 17% of cases (26) while El Karoui et al. found a more favorable evolution for patients with renal dysfunction (27). Histological lesions of TMA were associated with favorable renal prognosis (27). In our study, outcomes tended to be worse when hemodialysis was necessary (44% of deaths compare to 5% when hemodialysis was not needed), without differences in outcome between cases described as MPGN and TMA-like.

The pathogenesis of renal complications of patients with iMCD is not clear. Dysregulated IL-6 production and increased VEGF are known to play an important part in the development of iMCD and may be involved in the renal complications as well (26). Of note, VEGF is produced by podocytes in the glomerular endothelium for their maintenance (2, 32, 33). Glomerular endothelial cell fenestrations, which are induced by VEGF, are necessary for the permeability of the glomerular filtration barrier. Decreasing VEGF expression from the glomerulus causes a loss of fenestration and leads to microvascular injury and TMA even in the presence of increased circulating VEGF (33).

In iMCD, El Karoui et al. proposed a physiopathology model which could explain TMA lesions (27). VEGF glomerular expression (negatively correlated with the plasma CRP level, a marker of iMCD activity) was decreased. A possible cause for the decreased expression is the high circulating levels of IL-6 and VEGF (themselves induced by lymphoproliferation), which downregulate glomerular VEGF by podocytes via a negative-feedback mechanism.

Renal TMA has also been found in patients receiving anti-VEGF agents, and in those with preeclampsia (27, 33). These TMA lesions described are similar to the cases we present in this article (34).

Importantly, VEGF has been also proposed to play a key role in the pathogenesis of TAFRO syndrome. VEGF expression in iMCD patients with TAFRO syndrome seems, like other iMCD patients, upregulated (available VEGF level elevated in 11/12 cases) and yet, the patients have similar renal TMA lesions to those undergoing anti-VEGF therapy. Alternatively, in preeclampsia, for which level VEGF was habitually low, patients with the most severe cases of TMA have high VEGF levels (35). VEGF whether low or high, seems to contribute to renal involvement. Indeed, mice with loss function mutations in VEGF developed endotheliosis and “bloodless glomeruli,” and mice with overexpression of VEGF also had end-stage renal failure due to a collapsing glomerulopathy (capillary loops larger in diameter, multiple endothelial cell nuclei visible within and presence of mesangial cells in a crescent shape at the periphery of the glomerulus) (36). Another study in mice found that overexpression of VEGF formed new capillaries frequently containing portions of endothelium with pores without diaphragm and, stimulated the proliferation of fibroblasts (7 cases of glomerulosclerosis in our study) (37). This may occur because VEGF overexpression appears to initiate a feedback-mediated decrease in VEGF production by podocytes. This combination of increased serum levels but decreased local production of VEGF could explain the TMA glomerular injury found in patients with TAFRO syndrome (37), but further research is needed.

The physiopathology of MPGN renal lesions is even less well-understood than TMA-like lesion. Histologically, TMA is known to encompass a large range of changes including chronic forms in which lesions are similar to MPGN (34). In our study, most of MPGN cases presented with mild mesangial proliferation, which is certainly consistent with the presence of an underlying chronic TMA. VEGF is also known to induce PDGF-B production by endothelial cells (and possibly other mediators) that stimulates mesangial cell proliferation (38). Moreover, elevated IL-6 is thought to cause an increase in proliferation of renal mesangial cells (39), MPGN lesions (40), and VEGF production (5). We speculate that there is a continuum between TMA-like and MPGN renal lesions in TAFRO syndrome that may involve VEGF, PDGF-B, and IL-6 production.

Moreover, prerenal dysfunction provoked by vascular permeability, due to elevated VEGF, and hypoalbuminemia might also contribute to worsening renal function (41–43). Similar to Ito et al. we found endocapillary macrophage infiltration in our case; activated macrophages are key producers of both IL-6 and VEGF (5).

In conclusion, we present a Caucasian patient with TAFRO syndrome clinical subtype of iMCD who had renal insufficiency secondary to MPGN, probably also secondary to a chronic TMA. A literature review of all TAFRO syndrome cases wherein a renal biopsy was performed and our case (*n* = 20) highlights patients with a renal histopathology encompassing both TMA-like lesions and MPGN. Contrary to the heterogeneity of renal histology across all forms of CD, the renal histology of TAFRO syndrome cases was consistent with only two renal pathologies and with a great deal of overlap between each other. We suggest that there is a continuum between TMA and MPGN lesions in TAFRO syndrome that could explain why there does not seem to be any obvious difference in their clinical and biological presentation, renal histopathological features, and their therapeutic management. More research is needed to elucidate the roles that VEGF and IL-6 might be playing in the renal involvement identified in TAFRO syndrome.

## Data Availability

All datasets generated for this study are included in the manuscript and/or the supplementary files.

## Ethics Statement

This patient provided informed written consent for this publication.

## Author Contributions

All authors were involved in drafting the article or revising it critically for important intellectual content and approved the final version to be published.

### Conflict of Interest Statement

DF receives research funding from Janssen Pharmaceuticals. The remaining authors declare that the research was conducted in the absence of any commercial or financial relationships that could be construed as a potential conflict of interest.
